# Parkinson’s Disease: A Systemic Inflammatory Disease Accompanied by Bacterial Inflammagens

**DOI:** 10.3389/fnagi.2019.00210

**Published:** 2019-08-27

**Authors:** Büin Adams, J. Massimo Nunes, Martin J. Page, Timothy Roberts, Jonathan Carr, Theo A. Nell, Douglas B. Kell, Etheresia Pretorius

**Affiliations:** ^1^Department of Physiological Sciences, Faculty of Science, Stellenbosch University, Stellenbosch, South Africa; ^2^Department of Biochemistry, Institute of Integrative Biology, Faculty of Health and Life Sciences, University of Liverpool, Liverpool, United Kingdom; ^3^Division of Neurology, Department of Medicine, Faculty of Medicine and Health Sciences, Stellenbosch University, Stellenbosch, South Africa

**Keywords:** Parkinson’s disease, systemic inflammation, cytokines, LPS from *Porphyromonas gingivalis*, gingipains, amyloid formation

## Abstract

Parkinson’s disease (PD) is a well-known neurodegenerative disease with a strong association established with systemic inflammation. Recently, the role of the gingipain protease group from *Porphyromonas gingivalis* was implicated in Alzheimer’s disease and here we present evidence, using a fluorescent antibody to detect gingipain R1 (RgpA), of its presence in a PD population. To further elucidate the action of this gingipain, as well as the action of the lipopolysaccharide (LPS) from *P. gingivalis*, low concentrations of recombinant RgpA and LPS were added to purified fluorescent fibrinogen. We also substantiate previous findings regarding PD by emphasizing the presence of systemic inflammation via multiplex cytokine analysis, and demonstrate hypercoagulation using thromboelastography (TEG), confocal and electron microscopy. Biomarker analysis confirmed significantly increased levels of circulating proinflammatory cytokines. In our PD and control blood analysis, our results show increased hypercoagulation, the presence of amyloid formation in plasma, and profound ultrastructural changes to platelets. Our laboratory analysis of purified fibrinogen with added RgpA, and/or LPS, showed preliminary data with regards to the actions of the protease and the bacterial membrane inflammagen on plasma proteins, to better understand the nature of established PD.

## Introduction

Parkinson’s disease is a neurodegenerative disease caused by the death of dopaminergic neurons in the substantia nigra pars compacta (SNpc), resulting in dopamine deficiency within the basal ganglia. This can lead to a movement disorder with classical parkinsonian motor symptoms, as well as other symptoms. Although a number of Park genes have been identified (Funke et al., 2013), 90% of PD cases have no identifiable genetic cause (Klein and Westenberger, 2012; Ascherio and Schwarzschild, 2016). PD has a multitude of pathologies (Fujita et al., 2014), ranging from mis-folding of α-synuclein to neuro-inflammation, mitochondrial dysfunction, and neurotransmitter-driven alteration of brain neuronal networks (Titova et al., 2017); as well as affecting all levels of the brain-gut axis (Mulak and Bonaz, 2015).

**FIGURE 1:**
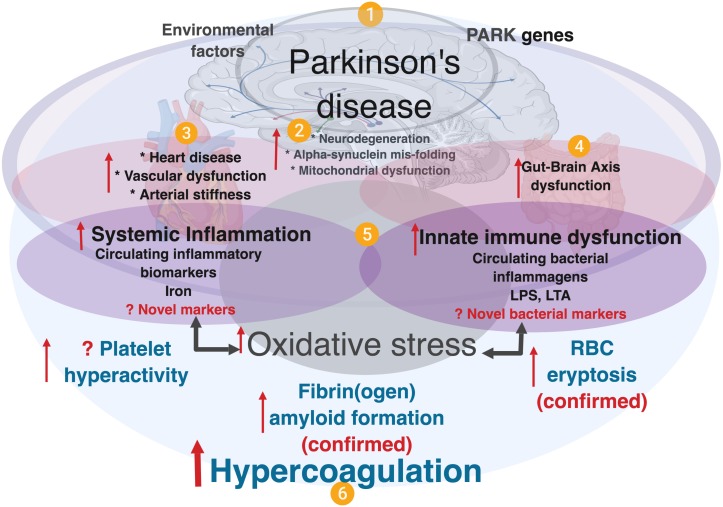
A simplified diagram showing contributing factors in systemic inflammation and hypercoagulation in Parkinson’s disease. **(1)** PD is characterized by the presence of PARK genes, and driven by environmental factors with **(2)**, neurodegeneration, and accompanied by **(3)** heart and vascular dysfunction, and also **(4)** gut-brain dysfunction. In PD, dysregulated inflammatory biomarkers and increased circulating bacterial inflammagens (e.g., LPS and LTA), point to **(5)** the presence of systemic inflammation and a dysfunctional innate immune system. Systemic inflammation is usually accompanied by oxidative stress that typically causes a general hypercoagulable state **(6)**, visible as platelet hyperactivity, RBC eryptosis and fibrin(ogen) amyloid formation. Diagram created using BioRender (https://biorender.com/).

Neuro-inflammation is an important and well-known feature of PD pathology (More et al., 2013; Nolan et al., 2013; Taylor et al., 2013), and converging evidence further supports the roles of (systemic) inflammation, oxidative stress (Kalia and Lang, 2015) and gut dysbiosis, although the mechanistic details and their full roles in PD pathogenesis are yet to be comprehensively elucidated. It is also noted that there are higher levels of proinflammatory cytokines in brains of PD patients, and inflammation is thought to be a major contributor to the neurodegeneration (Reale et al., 2009). Refer to Figure 1 for an explanatory overview of PD etiology and our interpretation of the role of systemic inflammation and (hyper)coagulation in this condition.

PD patients suffer from a plethora of other (inflammatory) comorbidities (Kell and Pretorius, 2018a), and both vascular risk (Cheng et al., 2017) and cardiovascular autonomic dysfunction are associated with arterial stiffness and heart disease (Pilotto et al., 2016; Kim et al., 2017). While the interplay between inflammation and neuronal dysfunction is complex, there is mounting evidence that chronic inflammation (Pretorius et al., 2014) with the accompanying dysregulation of circulating inflammatory molecules and the innate immune response, play prominent roles in PD (Kannarkat et al., 2013). It is also becoming recognized that peripheral, as well as brain inflammation, contribute to the onset and progression of the neurodegenerative processes seen in PD (Deleidi and Gasser, 2013; More et al., 2013; Nolan et al., 2013; Taylor et al., 2013; Filiou et al., 2014; Pessoa Rocha et al., 2014).

Evidence of systemic inflammation in PD includes the presence of increased levels of circulating cytokines such as IL-1β, IL-2, IL-10, IL-6, IL-4, TNF-α, C-reactive protein, RANTES, and interferon-gamma (INF-γ) (Brodacki et al., 2008; Qin et al., 2016). These markers are accompanied by oxidative stress and might even provide early diagnosis of PD (Lotankar et al., 2017). Recently Rathnayake et al. (2019) evaluated the role of selected serum immune mediators (i.e.,) IFNγ, TNFα, IL10 and NOX in PD progression and estimated their usefulness in preclinical diagnosis. They showed that IFNγ and IL-10 are involved in disease severity and that TNFα-mediated neurotoxicity appears to occur in early PD. In a meta-analysis, aberrations in peripheral cytokine levels were hypothesized to be related to PD, and the authors concluded that higher peripheral concentrations of IL-6, TNFα, IL-1β, IL-2, IL-10, C-reactive protein, and RANTES in patients strengths the clinical evidence that PD is accompanied by an inflammatory response (Qin et al., 2016).

In addition to dysregulated circulating inflammatory molecules, one of the known hallmarks of systemic inflammation is hypercoagulability, or abnormal clotting potential. In PD, changes in the normal clotting of blood have been described (Sato et al., 2003; Rosenbaum et al., 2013; Pretorius et al., 2014, 2018c; Infante et al., 2016; de Waal et al., 2018). Most of these circulating inflammatory biomarkers act as ligands to receptors on platelets (Olumuyiwa-Akeredolu et al., 2019), resulting in downstream signaling events with accompanying platelet hyperactivity and aggregation. RBCs also become eryptotic (programed cell death in RBCs) due to ligand binding and oxidative stress (Pretorius et al., 2014).

What remains unclear is the actual origin of the inflammation, how and why it is chronic. For this and other diseases (Potgieter et al., 2015; Kell and Kenny, 2016; Pretorius et al., 2016a, 2017a; de Waal et al., 2018; Kell and Pretorius, 2018a, b) we have gathered evidence that the main cause may be (dormant) microbes that upon stimulation, especially with unliganded iron (Kell, 2009), can briefly replicate and shed potent (and well known) inflammagens such as lipopolysaccharide (LPS) and LTA (Kell and Pretorius, 2015, 2018a). These are well-known ligands for receptors such as Toll-like receptor 4 (TLR4) that can activate inflammation, as observed through a variety of inflammatory cytokines (Olumuyiwa-Akeredolu et al., 2019).

Another set of (novel) bacterial inflammagens that might cause damage to fibrin(ogen) proteins when present in circulation is represented by a group of proteases synthesized by *Porphyromonas gingivalis*. *P. gingivalis* is a Gram-negative anaerobic bacterium that is deemed a keystone pathogen in the oral cavity with the capacity to shift symbiotic homeostasis into a dysbiotic state characteristic of periodontal pathogenesis (Darveau et al., 2012; How et al., 2016). Accordingly, this bacterium is significantly associated with and demonstrated to be a cause and driver of chronic periodontitis – the most common oral disorder among adults (Nazir, 2017). The bacterium’s entry into the circulation has been well-documented (Silver et al., 1977; Parahitiyawa et al., 2009; Tomas et al., 2012; Ambrosio et al., 2019); where it enters mainly through teeth care activities and oral ulcerations. Periodontal pathologies are known to be linked to systemic inflammation (Hajishengallis, 2015; Leira et al., 2018; Torrungruang et al., 2018), and *P. gingivalis* in particular is associated with a cohort of diseases including non-insulin dependent diabetes mellitus (Makiura et al., 2008; Blasco-Baque et al., 2017), Alzheimer’s disease (Singhrao et al., 2015; Dominy et al., 2019), rheumatoid arthritis (Okada et al., 2013; Mikuls et al., 2014; Jung et al., 2017), cardiovascular disease (Deshpande et al., 1998; Aarabi et al., 2015; Chistiakov et al., 2016; Leira et al., 2018) and atherosclerotic vascular tissue (Deshpande et al., 1998; Velsko et al., 2014; Olsen and Progulske-Fox, 2015).

The bacterium mainly uses oligopeptides as its energy substrate that are obtained via protease activities. Recently, emphasis was placed on both the bacterium and its group of endogenous cysteine proteases, known as gingipains, in developing Alzheimer’s disease, where gingipain was implicated in disease causation and suggested as possible disease intervention targets (Dominy et al., 2019). Gingipain is an important protease of *P. gingivalis* and its proteolytic activity plays an important part of the functioning of the bacterium, as it is essential for obtaining nutrients via protein degradation, adherence to host surfaces and further colonization (Guo et al., 2010). This protease is also known to play an important role in neutralizing the host defenses by degrading antibacterial peptides (Guo et al., 2010), and interfering or evading the host complement system (Slaney and Curtis, 2008). These enzymes cleave proteins at the C-terminal after arginine or lysine residues and are classified accordingly: gingipain R which is arginine-specific, and gingipain K lysine-specific.

There are two types of arginine-specific gingipains: RgpA, which seems to be the more virulent (Imamura et al., 2000) and RgpB. Not only are gingipains found on the cell surface of *P. gingivalis*, but are also secreted from the bacterium and can thus enter the circulation, where it may interact with various circulating blood proteins, including clotting proteins. Studies have demonstrated fibrinogen-adhesive and fibrinogenolytic effects arising from each gingipain type (Lantz et al., 1986; Imamura et al., 1995; Pike et al., 1996; Ally et al., 2003). Further, the effect of gingipain proteases on fibrinogen increases the propensity for bleeding at periodontal sites (symptom of periodontitis) thereby enabling *P. gingivalis* access to nutrient sources (heme-containing proteins) and inadvertently the circulation. The interference of these proteases in coagulation may not be exclusive to fibrinogen, and interactions have been shown with factor IX prothrombin (Imamura et al., 2001), factor X (Imamura et al., 1997) and prothrombin (Imamura et al., 2001), as well as the stimulation of the kallikrein–kinin pathway (Imamura et al., 1994). This pathway includes coagulation factor XII, the complex of prekallikrein and high molecular weight kininogen; and when this pathway is activated, it leads to the activation of several sequential effector proenzymes resulting in the induction of genes and activation of biomolecules involved in the molecular mechanisms of vasodilation, blood coagulation, and fibrinolysis (Bryant and Shariat-Madar, 2009).

Since periodontitis predisposes an individual to an exaggerated risk of developing PD (Kaur et al., 2016; Chen et al., 2017, 2018), and because the activity of *P. gingivalis* and gingipains have recently been highlighted in Alzheimer’s patients (Dominy et al., 2019), we speculate that this bacterium and its molecular products (e.g., proteases and LPS) might be present in the circulation of PD individuals.

In this paper, we therefore aim to offer further evidence of significant systemic inflammation and the presence of circulating inflammagens in PD compared to controls. Here, we provide evidence on the extent of the dysregulated systemic inflammatory biomarker profile, hypercoagulability and particularly platelet hyperactivity in PD patients compared to healthy individuals, and how dysregulated inflammatory circulating molecules could, in part, be responsible for blood hypercoagulability and platelet dysfunction. Additionally, we examine whole blood clot formation using thromboelastography, and view platelet ultrastructure using scanning electron microscopy. Furthermore, we hypothesize how these dysregulated inflammatory molecules might act as ligands when they bind to platelet receptors, resulting in activation of platelet signaling cascades. We argue that the levels of inflammatory molecule dysregulation point to innate immune system activation, which is supportive of our previous published results regarding the presence of LPS in/near hypercoagulated blood clots (de Waal et al., 2018). We confirm the presence of amyloid fibrin(ogen) in the current sample, using amyloid-specific markers [previously we used only ThT as a marker of aberrant clotting in PD (Pretorius et al., 2018a)]. To date, *P. gingivalis* and its molecular signatures are yet to be discovered in PD tissue (other than the oral cavity). We present evidence (using fluorescent antibodies against gingipains), that members of the gingipain protease family are present in clots from PD samples, but not significantly present in healthy plasma clots. We also add purified RgpA to purified fibrinogen marked with a fluorescent Alexa 488 marker, and show how it potentially can hydrolyze fibrinogen proteins and that gingivalis LPS may act together with gingipains to foster aberrant clot formation (see Supplementary Figure 1 for a layout of our experiments).

## Materials and Methods

### Ethical Clearance and Consent

The study received ethical clearance from the Health Research Ethics Committee (HREC) of Stellenbosch University, South Africa (HREC Reference # S18/03/054) and the Health Department of Western Cape research number (WC_201805_023). Written informed consent was obtained from all participants followed by whole blood sampling. All participants received a unique number that was used to guarantee discretion throughout this study. All investigators were certified in Good Clinical Practice and ethical codes of conduct.

### Study Design, Setting, and Study Population

Blood samples were collected from both healthy and PD patients residing in the greater Stellenbosch/Boland region of the Western Cape South Africa. The PD patients visited Tygerberg hospital for specialist primary health care treatment. Age-matched healthy individuals (confirmed not having PD) were recruited from the same geographical region. A cross-sectional design was followed in collaboration with a neurologist, that provided WB from PD patients. Whole blood from healthy controls was collected by a Health Professions Council of South Africa (HPCSA) registered Medical Biological Scientist and phlebotomist (MW: 0010782) at the Department of Physiological Sciences, Stellenbosch University. A total of *n* = 81 volunteers were included (*n* = 41 healthy controls, and *n* = 40 PD patients) as part of the study population. PD patients were recruited with the following inclusion criteria: (i) a confirmed diagnosis by a neurologist using the Unified Parkinson’s Disease Rating Scale (UPDRS), as well as the Hoehn and Yahr scale to rate the relative level of the PD disability, (ii) males and females of any age, (iii) not on any anticoagulant medication. Participants who were unable to provide written consent were ineligible for the study. To limit and exclude confounding factors, both healthy and PD volunteers were only recruited and included if they were not diagnosed with tuberculosis, HIV or any malignancies. The inclusion criteria for healthy age-matched volunteers included were: (i) no use of chronic medication for inflammation, (ii) no prior history of thrombotic disease (stroke/heart attack) or serious inflammatory conditions, (iii) non-smokers, (iv) not on any chronic antiplatelet therapy/anticoagulant medication or any contraceptive/hormone replacement therapy, (v) not pregnant and/or lactating. PD is a progressive condition which tends to evolve from mild unilateral symptoms through to an end-stage non-ambulatory state (refer to demographic Table 1; and Supplementary Tables 1,2 for the milestones in the illness as accurately outlined in the Hoehn and Yahr staging system).

**TABLE 1:** Demographics, Hoehn and Yahr scale and medicine use for control and PD patients.

	**Controls** **(*n* = 41)**		**Parkinson’s** **(*n* = 40)**					
Age (years)	59 [53.5–72.0]		66 [62.3–72.0]					
Gender distribution	*n* = 26 (females); *n* = 15 (males)		*n* = 15 (females); *n* =25 (males)					
Hoehn and Yahr Scale	*n* = 0	1	1.5	2	2.5	3	4	5
		*n* = 7	*n* = 1	*n* = 13	*n* = 6	*n* = 9	*n* = 3	*n* = 1
Cholesterol medication^1^ (n)	*n* = 3 (Aspavor^®^)		*n* = 1 (Simvastatin^®^)					
Blood pressure medication^2^ (n)	*n* = 13 (Bisoprolol, Natrilix, Prexum^®^)		*n* = 14 (Lasix^®^, hydrochlorothiazide, Enalapril^®^, Amlodipine^®^)					
Diabetes medication (n)	*n* = 0		*n* ( =8 (Metformin^®^, Lantis^®^)					
Parkinson’s medication (n)	*n* = 0		*n* = 40 (Cabilev^®^, Levodopa, Donepeidon^®^, Requip^®^, Pexola^®^, Sinemet^®^, Rivotnol^®^)					

*^1^Both belong to statin class, ^2^Prexum and Enalapril (ACE inhibitors) Bisoprolol – beta blocker, Natrilix and Lasix similar in mechanism HTCZ – diuretic, Amlodipine – Ca^*2*+^ blocker.*

### Collection of Whole Blood (WB) and Preparation of Platelet Poor Plasma (PPP) Samples From Healthy Controls and PD Patients

Whole blood from healthy controls and PD patients were collected using sterile sampling techniques in citrated, EDTA and SST tubes that were kept at room temperature (∼22°C) for 30 min. This is within the prescribed manufacturer protocol for blood collection. PPP was prepared from citrate tubes that were centrifuged at 3000 × *g* for 10 min at room temperature (∼22°C). The PPP was subsequently aliquoted into labeled 1.5 mL Eppendorf tubes, and stored at −80°C until cytokine analysis. EDTA whole blood and SST were analyzed by the local PathCare laboratory (Stellenbosch) for HbA1c, TC, low-density lipoprotein cholesterol (LDL-c), high-density lipoprotein cholesterol (HDL-c), TG and non-high-density lipoprotein (non-HDL); TC/HDL ratio was calculated as a marker of cardiovascular risk.

### Thromboelastography (TEG) of Whole Blood (WB) and 20-Plex Cytokine Analysis Using Platelet Poor Plasma (PPP)

Clot kinetics/property analysis was completed by means of TEG (Thromboelastograph 5000 Hemostasis Analyzer System, Haemonetics S.A. Signy-Avenex, Switzerland), on both control and PD WB samples. 340 μL of WB samples were placed in a disposable TEG cup to which 20 μL of 0.2 mol/L CaCl_2_ was added. CaCl_2_ is necessary to reverse the effect of the sodium citrate anticoagulant in the collection tube (i.e., recalcification of blood) and consequently initiate coagulation.

Stored PPP samples of PD (*n* = 40) and healthy controls (*n* = 41) participants were transferred from −80°C to −20°C 24 h preceding the multiplex analysis. The samples were then analyzed in duplicate by means of Invitrogen’s Inflammation 20-Plex Human ProcartaPlex^TM^ Panel (#EPX200-12185-901) and read on the Bio-Plex^®^ 200 system (Bio-Rad). 20 anti- and pro-inflammatory molecules were measured in a multiplex analysis and biomarkers measured included 4 anti-inflammatory molecules (IFN-α, IL-4, IL-10, IL-13), and 16 pro-inflammatory molecules (for the full list of cytokines, refer to see section “Results”).

Three variations of logistic regression modeling were investigated to determine the strength of association between the measured parameters and PD status. For all three models, odds ratios (OR) with 95% confidence intervals, calculated via profiling, are reported in a manner that allows inter-model comparison. Logistic regression was performed between Parkinson’s status (binary) and all individual parameters both with no adjustment (Model 1) and with adjustment for age and gender (Model 2). Ordinal logistic regression was performed between the Hoehn and Yahr severity scale and all individual parameters (Model 3) to determine which parameters are associated with disease progression. The ordinal model was calculated without adjustment due to sample size requirements.

Three individuals (2 control, 1 PD) were withheld from this statistical analysis due to missing data leaving 39 observations in both groups. Statistical analysis was performed using R version 3.5.3 using glm for logistic regression and clm for ordinal regression. Mann Whitney non-parametric tests were performed and contrasted with the results from logistic regression. Although the Mann Whitney test was found to be *more sensitive* (identifying a super-set of parameters as significant), the logistic regression model was deemed more appropriate due to (a) the ability to perform adjustment and ordinal modeling and (b) providing *more conservative* results in the context of the outliers due to the method identifying monotonic trends instead of median differences. One could also plausibly argue, based on the Box and Whisker plots shown later in the results section, that the requirement of Mann Whitney for the same data distribution are not upheld (different skews and outlier levels). PCA analysis was performed using the prcomp method from the built-in stats package.

### Scanning Electron Microscopy of Whole Blood (WB) Smears

Whole Blood smears were prepared by placing 10 μL WB of each of the samples on cover slips. Samples were washed with Gibco^TM^ PBS, pH 7.4 (ThermoFisher Scientific, 11594516) before fixing with 4% paraformaldehyde for a minimum of 30 min. Once fixed, samples were washed 3 × 3 min with PBS followed by a second 30-min fixation step in 1% osmium tetroxide (Sigma-Aldrich, 75632). A final 3 × 3 min PBS wash step was performed before samples were serially dehydrated in ethanol with a final 30-min dehydration step using hexamethyldisilazane (HMDS) ReagentPlus^®^ (Sigma-Aldrich, 379212). Samples were then carbon coated before being imaged on Zeiss MERLIN^TM^ field emission scanning microscope and micrographs were captured using the high resolution InLens capabilities at 1 kV.

### Recombinant Gingipain R1 Protease (RgpA) and Gingipain R1 Antibody

Platelet poor plasma (PPP) was used to prepare clots for imaging from PPP from *n* = 30 healthy and *n* = 34 PD samples. Thrombin was donated by the South African National Blood Service; it was solubilized in PBS containing 0.2% human serum albumin to obtain a concentration of 20 U.mL^–1^ and was used at a 1:2 ratio to create extensive fibrin networks. This was followed by fixation with 10% NBF. After PBS (pH = 7.4) washing steps, samples were blocked with 5% goat serum (in PBS), and incubated with gingipain R1 polyclonal antibody (Abbexa, abx 107767; 1:100 in 5% goat serum) for 1 h at room temperature in the dark. The samples were finally washed and a coverslip was mounted with a drop of Dako fluorescence mounting medium on a microscopy slide for confocal analysis. The prepared samples were viewed on a Zeiss LSM 780 with ELYRA PS1 confocal microscope using a Plan-Apochromat 63×/1.4 Oil DIC objective. The gingipain R1 FITC antibody was excited at 488 nm, with emission measured at 508 to 570 nm. As a positive control, we also incubated an exogenous aliquot of the protease, recombinant gingipain R1 protease (RgpA), with healthy PPP for 30 min, followed by exposure to its fluorescent antibody. RgpA (Abcam ab225982) was added at a final concentration of 500 ng L**^–^**^1^. To assess the association between the presence of gingipain R1 and PD, the pixelwise mean of the (green) RgpA immunofluorescence channel was extracted from images of both populations. Multiple images were acquired and analyzed per participant due to the spatial variability inherent in this image-based approach. A logistic regression was performed between PD status and the mean channel intensity over all acquire images.

### Recombinant Gingipain R1 Protease and Alexa 488-Conjugated Purified Fibrinogen

Purified (human) fibrinogen with Alexa 488 (ThermoFisher: F13191) was prepared to a final concentration of 2 mg.mL**^–^**^1^. Clots (with and without the protease, RgpA) were prepared by adding human thrombin as per the above protocol. Clots were also viewed with the confocal microscope and fluorescent fibrinogen was excited at 488 nm, with emission measured at 508 to 570 nm. As the gingipains antibody used above has the same excitation and emission as the purified fibrinogen, we could not trace the added gingipains with this antibody. We also incubated purified fluorescent fibrinogen with LPS from *P. gingivalis* (10 ng L^–1^) with and without RgpA (both 100 and 500 ng L**^–^**^1)^. Where we combined the LPS and the RgpA, both were added simultaneously followed by an incubation period of 30 min.

### Confocal Analysis of Plasma Clots to Show Amyloid Fibrin(ogen)

To show amyloid formation of blood plasma, that might be one of the causes of hypercoagulation, we analyzed amyloid presence using three fluorescent amyloid markers. These markers were added to control and PD PPP to illuminate amyloid protein structure, and were used as follows: 5 μM ThT, 0.1 μL (stock concentration as supplied) of AmyTracker 480 and 0.1 μL (stock concentration as supplied) of AmyTracker 680 were added to the sample to incubate for 30 min. A working solution of AmyTracker was made in PBS at a 1:20 ratio. Control and PD PPP clots were prepared by adding thrombin to activate fibrinogen and create extensive fibrin fiber networks. Using the same microscope and objective as above, three channels were setup to visualize the amyloid markers. Amytracker 480 was excited by the 405 nm laser, with emission measured at 478 to 539 nm; Amytracker 680 was excited by the 561 nm laser, with emission measured at 597 to 695 nm; and ThT was excited by the 488 nm laser, with emission measured at 508 to 570 nm. ThT may also be excited by the 405 laser, and has a wide spectra where fluorescence can be detected (Sulatskaya et al., 2017). We allowed these two stains, which both target amyloid structures, to overlap in the microscope setup to produce a combination blue channel of amyloid signal, alongside the isolated signal from Amytracker 680 in the red channel and ThT in the green channel (Page et al., 2019). Micrographs of the prepared clots were captured as 3 × 3 and 2 × 2 tile images, and 75 images from *n* = 25 PD patients, and 39 images from *n* = 9 control donors were acquired. Gain settings were kept constant for all data acquisition and used for statistical analyses, however, brightness and contrast were adjusted for figure preparation. The mean and the standard deviation from the histogram of each image were recorded and used to calculate the coefficient of variance (CV), which is defined as SD ÷ mean. This metric was used to quantify and discriminate the relative variation between the signal present between control and PD PPP clots. CVs of the healthy control and PD group were compared by the Mann–Whitney test in GraphPad Prism 7.04 with significance accepted at *p* < 0.05.

## Results

Tables 2A,B provide summary statistics and results from all three regression models for markers from WB for healthy controls and PD patients. More specifically, Table 2A indicate the seven TEG clotting parameters as well as lipid profile, HbA1c, and ultrasensitive CRP level, and Table 2B presents anti-inflammatory and pro-inflammatory cytokine markers. The three regression models consistently identify the same parameters as significant, at a level of 0.05 (identified as bold in the Tables 2A,B). The one exception is IL-1β, which was not significantly predictive in the ordinal logistic regression model (i.e., not predictive of the scale of the disease). One can also observe that significant markers exist across all groupings except anti-inflammatory markers. To summarize, the following parameters in each group can be identified as significantly different:

**TABLE 2A:** Thromboelastography results showing seven viscoelastic parameters assessing coagulation properties of healthy control and PD WB samples.

	**Controls** **(*n* = 39)**	**PD** **(*n* = 39)**	**Model 1 Unadjusted OR (95% CI)**	**Model 2 Adjusted OR (95% CI)**	**Model 3 Unadjusted ordinal OR (95% CI)**
**TEG Clot Parameters**					
*R*-value (min)	8.3 [7.15–9.85]	7.2 [6.35–8.35]	**0.678** **(0.503–0.875)**	**0.659** **(0.479–0.859)**	**0.753** **(0.591–0.938)**
*K*-value (min)	2.7 [2.2–3.05]	2.2 [1.8–3]	0.944 (0.604–1.46)	0.942 (0.568–1.52)	0.947 (0.631–1.38)
A Angle (°)	59.7 [53.1–63.95]	64.9 [59.75–70.25]	**1.08** **(1.02–1.16)**	**1.08** **(1.01–1.16)**	**1.07** **(1.01–1.13)**
MA (mm)	59.8 [54.35–63.9]	56.8 [49–59.75]	0.949 (0.894–1)	0.954 (0.896–1.01)	0.965 (0.924–1.01)
MRTG (Dyn. cm^–2^.s^–1^)	4.7 [4.17–5.85]	5.49 [3.96–6.7]	1.09 (0.869–1.39)	1.12 (0.861–1.47)	1.05 (0.858–1.29)
TMRTG (min)	12.3 [10.2–13.6]	9.7 [8.6–12]	**0.712** **(0.568–0.864)**	**0.7** **(0.544–0.865)**	**0.779** **(0.653–0.916)**
TTG (Dyn. cm^–2^)	745.5 [598–882]	658 [481–744]	0.998 (0.996–1)	0.998 (0.996–1)	0.998 (0.996–1)
**Lipogram Parameters**					
TC (mmol L^–1^)	5.6 [4.65–6.4]	5.2 [4.25–5.7]	0.856 (0.593–1.22)	1.09 (0.723–1.67)	0.819 (0.586–1.13)
HDL (mmol L^–1^)	1.5 [1.3–1.8]	1.3 [1.1–1.4]	**0.164** **(0.0363–0.583)**	**0.201** **(0.0394–0.816)**	**0.197** **(0.0487–0.662)**
Trig (mmol L^–1^)	1.08 [0.86–1.65]	1.32 [0.92–1.91]	0.898 (0.574–1.39)	1.2 (0.726–2)	0.83 (0.553–1.23)
LDL (mmol L^–1^)	3.2 [2.45–3.9]	3.2 [2.4–3.65]	1.29 (0.787–2.24)	1.49 (0.891–2.67)	1.12 (0.715–1.74)
HbA1c (%)	5.3 [4.8–5.6]	5.8 [5.5–6.25]	**8.49** **(3.02–32.8)**	**6.89** **(2.38–28.2)**	**1.94** **(1.28–3.1)**
Ultrasensitive CRP (mg L^–1^)	1.63 [0.53–2.88]	1.49 [0.715–4.06]	1.1 (0.956–1.3)	1.16 (0.995–1.42)	1.03 (0.924–1.15)

*Whole blood lipid profiles, Glycosylated Hemoglobin and ultrasensitive CRP. Data expressed as median and [25–75% quartile range]. Bold values indicate statistical significance.*

**TABLE 2B:** Anti-inflammatory and pro-inflammatory cytokine profiles of healthy and PD volunteers are also shown.

**Cytokines** **(pg mL**^–^**^1^)**	**Controls (*n* = 39)**	**PD (*n* = 39)**	**Model 1 Unadjusted OR (95% CI)**	**Model 2 Adjusted OR (95% CI)**	**Model 3 Unadjusted ordinal OR (95% CI)**
**Anti-inflammatory markers**					
IFN-α	0.61 [0.001–1.39]	0.001 [0.001–1.4]	0.845 (0.545–1.12)	0.88 (0.537–1.18)	0.838 (0.549–1.1)
IL-10	3.44 [1.98–8.28]	4.52 [2.89–5.925]	0.95 (0.838–1.06)	0.966 (0.845–1.08)	0.96 (0.851–1.07)
IL-13	2.51 [0.53–5.66]	3.61 [2.405–4.9]	0.985 (0.881–1.09)	0.994 (0.877–1.1)	0.985 (0.884–1.09)
IL-4	14.88 [6.185–25.7]	11.62 [2.84–25.5]	0.979 (0.952–1)	0.983 (0.954–1.01)	0.979 (0.954–1)
**Pro-inflammatory markers**					
E-Selectin	27752 [21085–38694]	25801 [19141–34851]	1 (1–1)	1 (1–1)	NA
GM-CSF	14.25 [0.001–52.]	26.23 [13.1–48.2]	0.997 (0.984–1.01)	0.997 (0.983–1.01)	0.997 (0.984–1.01)
IFN-γ	7.4 [2.57–14.9]	9.39 [6.74–14.3]	0.995 (0.954–1)	0.997 (NA-1)	0.994 (0.95–1)
IL-1α	3.1 [1.44–4.065]	4.7 [2.52–11.76]	**1.25** **(1.1–1.5)**	**1.26** **(1.1–1.53)**	**1.04** **(1.01–1.08)**
IL-1β	15.99 [10.13–32.225]	24.42 [21.52–30.3]	**1.05** **(1.01–1.09)**	**1.04** **(1–1.09)**	1.03 (0.997–1.07)
IL-12p70	17.39 [9.56–96.085]	72.12 [21.95–105.5]	1.01 (0.997–1.02)	1.01 (0.997–1.02)	1.01 (0.997–1.01)
IL-17A	1.18 [0.001–11.69]	14.98 [11.5–18.7]	**1.08** **(1.03–1.15)**	**1.09** **(1.03–1.16)**	**1.07** **(1.02–1.13)**
IL-6	8.43 [0.86–27.18]	24.67 [20.29–34.82]	1.01 (1–1.03)	1.01 (1–1.03)	1 (0.999–1.01)
IL-8	1.66 [0.001–10.135]	10.97 [6.98–23.415]	1.02 (1–1.05)	1.02 (1–1.04)	1 (0.997–1.01)
IP-10	16.51 [12.635–23.505]	18.19 [15.49–21.12]	0.996 (0.966–1.03)	1 (0.969–1.03)	0.994 (0.965–1.02)
MCP-1	39.26 [24.06–50.93]	32.01 [27.1–38.23]	0.986 (0.957–1.01)	0.982 (0.951–1.01)	0.992 (0.966–1.02)
MIP-1α	16.04 [3.64–59.2]	28.32 [16.1–72.6]	1.01 (0.997–1.02)	1.01 (0.995–1.02)	1.01 (0.995–1.02)
MIP-1β	39.9 [18.9–277]	131.06 [57.25–306.4]	1 (0.999–1)	1 (0.998–1)	1 (0.998–1)
sP-Selectin	13236 [7647–38887]	28241 [14132–62124]	1 (1–1)	1 (1–1)	NA
sICAM-1	42392.75 [25290–64595]	46241 [31981–69231]	1 (1–1)	1 (1–1)	NA
TNF-α	55.44 [31.5–98.8]	107.6 [72.6–137]	**1.01 (1–1.02)**	**1.01 (1–1.03)**	**1.01 (1–1.02)**

**Data expressed as median and [25*–*75% quartile range].* Bold values indicate statistical significance.*

• **TEG parameters:** R, Angle, TMRTG
• **Lipogram parameters:** HbA1c, HDL
• **Anti-inflammatory markers**: None
• **Pro-inflammatory markers**: IL-1α, IL-17A, TNF-α, IL-1β

**FIGURE 2:**
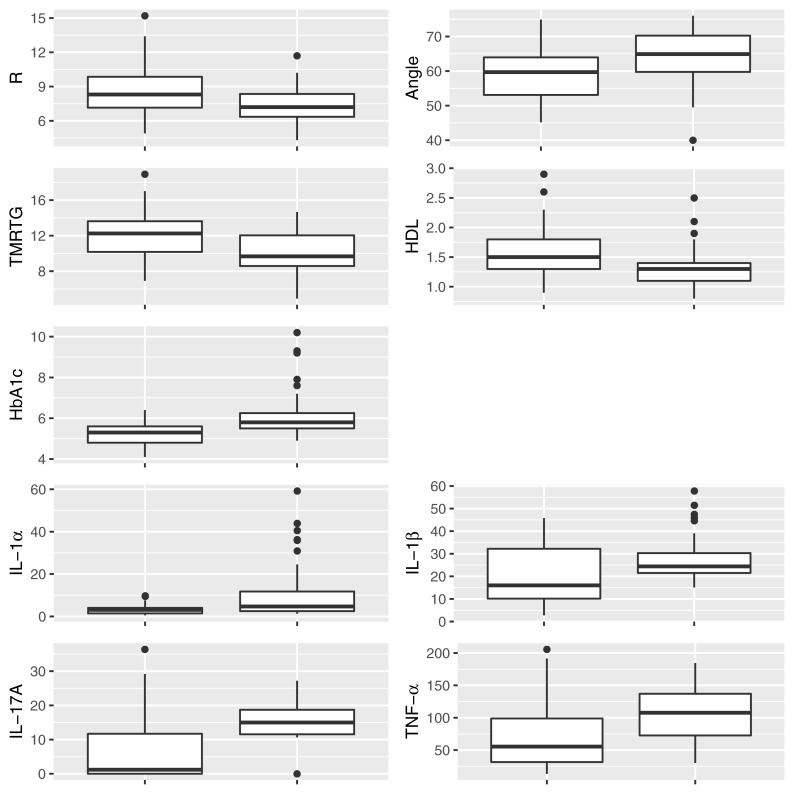
Box and whisker plots showing the distribution of parameters for control **(Left)** and PD **(Right)** populations for parameters determined to be significantly different.

**FIGURE 3:**
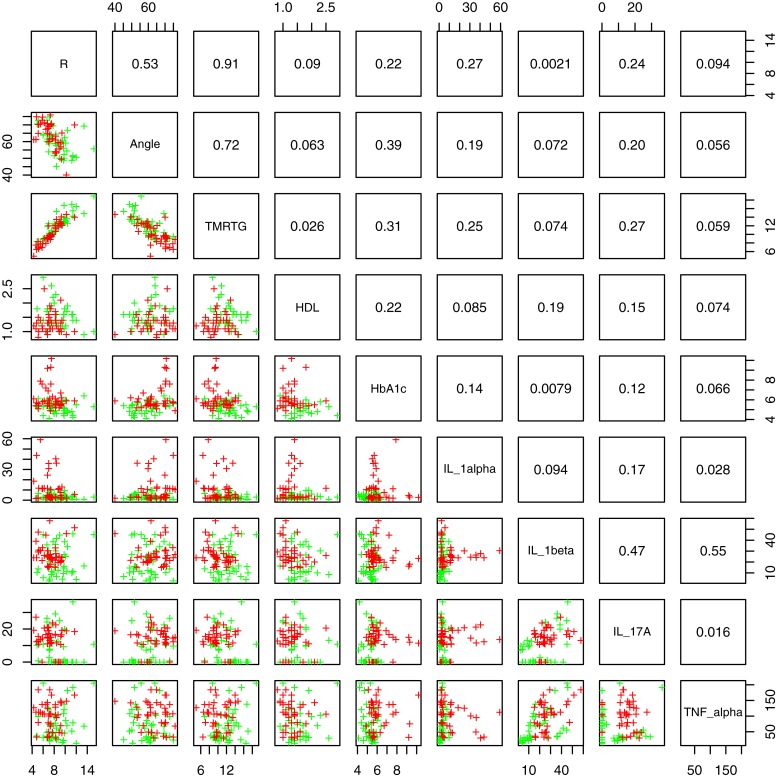
Lattice of cross-plots of statistically significant parameters colored by PD status (Green = Control). The upper diagonal shows correlation coefficients.

Figure 2 shows box and whisker plots for each of these significant parameters in order to illustrate the population distributions and highlight the presence of outliers. Figure 3 shows a lattice of cross-plots for these significant parameters along with correlation coefficients in the upper diagonal. From this one can observe, as would be expected, non-negligible correlations within the cytokine or TEG markers (e.g., between R and TMRTG). However, the intra-group correlations are low, suggesting that a combination of TEG and cytokine measurements would likely increase predictive power. Multivariate analysis of this form should be considered in future work with larger populations (Supplementary Figure 2 shows a visualization based on PCA analysis of the combined data. Ellipses for PD status are overlaid but were not part of the analysis. Notice that the first two principal components capture around 30% of the variance in the data).

### Thromboelastography, Cholesterol and HbA1c Levels, and Ultrasensitive CRP

HbA1c levels were significantly increased in the PD sample with a notably dysregulated lipid profile (see Table 2A). TEG results point to the fact that PD WB is hypercoagulable. TEG analysis exhibited significant differences in five of the groups of the assessed parameters. The PD group presented a significant increase in the initial rate of clot formation (*R*-value). Significant elevation in alpha angle (A angle) suggests more cross-linking of fibrin fibers, and time to maximum rate of thrombus generation (TMRTG) was decreased. These results have significance to our RgpA results that we discuss later.

### Scanning Electron Microscopy of Whole Blood

**FIGURE 4:**
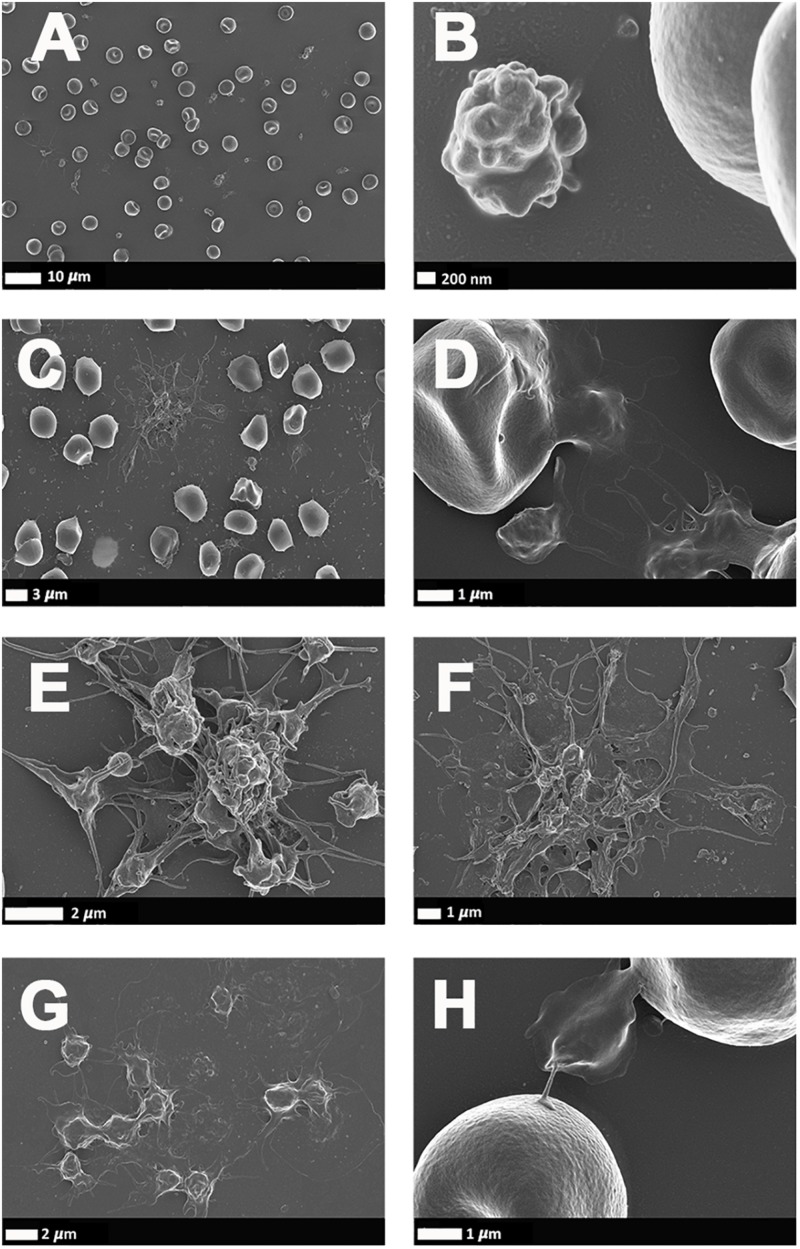
**(A,B)** Scanning electron microscopy of whole blood smears showing representative platelets from healthy individuals. **(C–H)** Whole blood smears from PD individuals showing hyperactivated platelets. **(C–H)** PD platelets agglutinating to RBCs; **(D,H)** PD platelet spreading **(G)** and PD platelet aggregation **(C,E,F)**.

Figure 4 illustrates a representative SEM micrographs of platelets seen in WB smears. SEM analysis of WB smears of healthy controls usually show platelets as irregularly shaped cellular structures, with only slight pseudopodia formation due to contact activation with glass cover slips. This finding has previously been noted in various publications (Pretorius et al., 2018d; Page et al., 2019, 2018). In the PD sample, platelets showed substantial (hyper)activation, spreading (Figures 4C,E–G), as well as aggregation (Figures 4F,G), suggesting that these results might be due to the increased inflammatory biomarkers and cytokines in circulation, that act as ligands to platelet receptors. Interactions with RBCs were also frequently noted (Figures 4D,H).

### The Analysis of Clots Formed From Fibrinogen Incubated With Recombinant Gingipain R1

**FIGURE 5:**
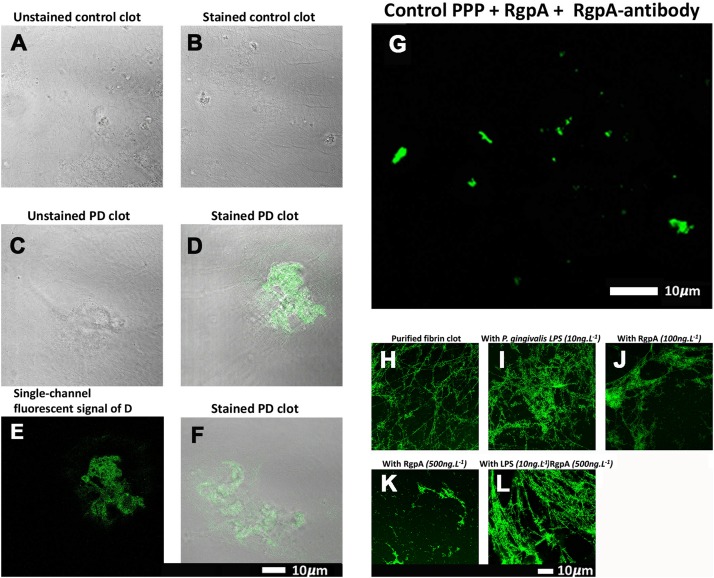
**(A–G)** Confocal microscopy images of PPP clots stained with the RgpA polyclonal antibody (1:100) from healthy individuals and individuals suffering from PD. The images **(A–G)** are two channel overlays where transmitted light microscopy micrograph and fluorescent signal are superimposed to show the areas of fluorescence on the clot itself, except **(E)** and **(G)** that show only the fluorescence signal. **(A)** The unstained and **(B)** stained control exhibits no fluorescent signal as well as **(C)** the unstained Parkinson’s disease PPP clots. **(D–F)** Fluorescent signal of the RgpA antibody is prominently detected in stained Parkinson’s disease PPP clots. **(G)** Represents a positive control in which a control sample that is absent of fluorescent signal received an exogenous load of RgpA. **(H–L)** Confocal microscopy images of fibrin networks formed from purified fibrinogen (with added Alexa488 fluorophore) incubated with and without RgpA, and LPS from *P. gingivalis*, followed by addition of thrombin to create extensive fibrin(ogen) clots. **(H)** Representative purified fibrin(ogen) clot. **(I)** A representative clot formed after purified fibrinogen was incubated with 10 ng L ^–1^ *P. gingivalis* LPS. **(J)** A representative clot formed after purified fibrinogen was incubated with 100 ng L ^–1^ RgpA and **(K)** 500 ng L ^–1^ RgpA. **(L)** A representative clot after purified fibrinogen was simultaneously exposed to a combination of *P. gingivalis* LPS (10 ng L ^–1^) and RgpA (500 ng L ^–1^).

Confocal microscopy was used to visualize the clot structure of purified fibrin(ogen) marked with Alexa 488, with and without exposure to recombinant gingipain R1 (500 ng L^–1^), and with and without exposure to *P gingivalis* LPS (Figures 5H–L). Note that fibrinogen was pre-incubated with the inflammagens,

followed by clot formation with thrombin. Figure 5H is a representative purified fluorescent fibrin(ogen) clot, showing a fibrin network with distinctive fibers. Figure 5I shows a representative fibrin(ogen) clot after fluorescent fibrinogen was incubated with *P. gingivalis* LPS. Fibrin networks display a denser and more matted network. Purified fibrinogen was also exposed to two concentration of RgpA (100 ng L**^–^**^1^) (Figure 5J) and 500 ng L**^–^**^1^ (Figure 5K). RgpA greatly inhibited fibrin formation synthesis in a concentration-dependent manner. A combination of both the LPS and RgpA (500 ng L**^–^**^1^) was also added simultaneously to purified fibrinogen, and the resulting clot is shown in Figure 5L. Interestingly, this clot appeared similar to the clot where only LPS was added (Figure 5I). Although the interactions between the various protease domains, and RgpA in particular, with LPS and their combined affects on fibrin(ogen) is unknown, LPS is known to bind to domains of

RgpA (Takii et al., 2005). Hence, this association may alter the capabilities of RgpA and may be a reason for the decreased RgpA effect on clot formation at 500 ng L**^–^**^1^ seen in the current results, with simultaneous LPS incubation. Therefore, we suggest that the LPS and the protease might function together, where the protease might hydrolyze the fibrin(ogen) peptides but the LPS might simultaneously cause aberrant coagulation.

### The Identification of Gingipain R1 in Parkinson’s Disease Blood Samples With Its Fluorescent Antibody

**FIGURE 6:**
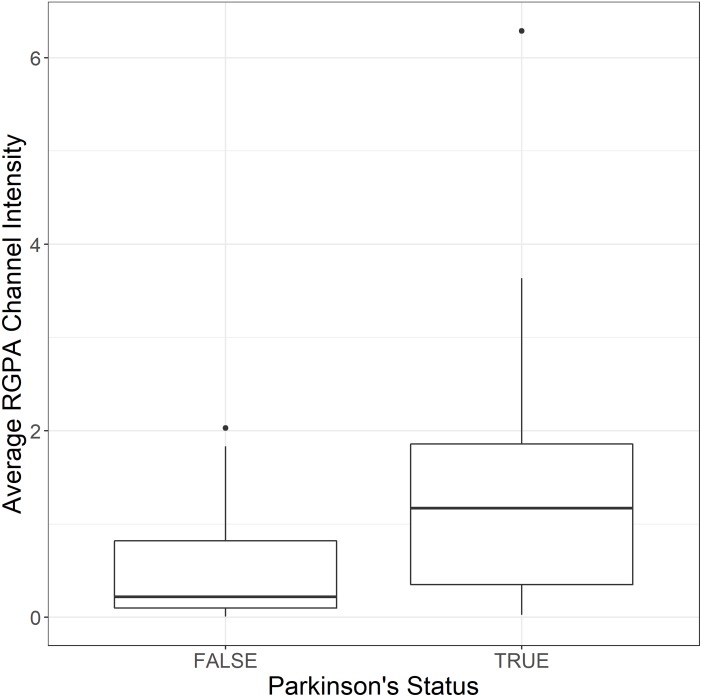
Box and whisker plot showing the distribution of mean RgpA image channel intensity for the healthy and PD populations.

To assess the association between the presence of gingipain R1 and PD, the pixelwise mean of the (green) RgpA immunofluorescence channel was extracted from images of

both populations. Three images were acquired and analyzed per participant due to spatial variability inherent in this image-based approach (note: ELISA assays for RGPA were not available at time of publication). A logistic regression model analyzing PD status versus image means showed a statistically significant association with a large odds ratio (OR) of 3.2 (1.6–7.5). Figure 6 illustrates this difference with a box and whisker plot. Again, we can see strong outliers in this univariate projection of the data. As positive control, we exposed controls to a tiny concentration of exogenous recombinant RgpA followed by polyclonal antibody staining against RgpA (Figure 5G). A distinct but minimal signal was now present. This was expected, as the concentration of RgpA added to healthy PPP was very low (500 ng L**^–^**^1^ final exposure).

### Confocal Analysis of Plasma Clots

**FIGURE 7:**
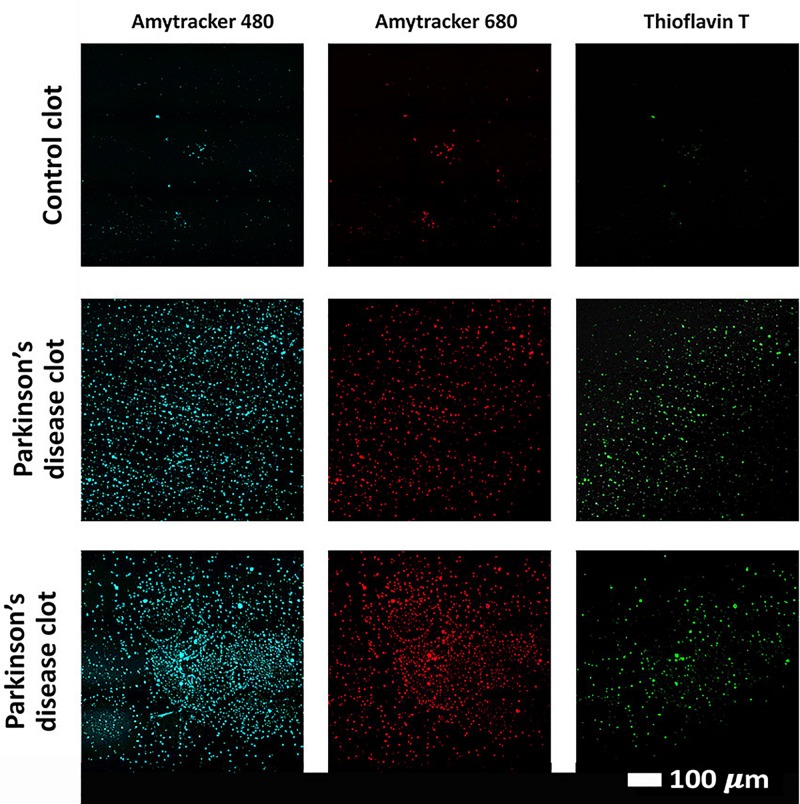
Examples of clots created with platelet poor plasma (PPP) for a representative control and two representative PD individuals to show amyloid fibrin(ogen) protein structure. Three fluorescent markers that bind amyloid protein were used, Amytracker 480, 680, and ThT (as previously used for amyloid fibrin structure (Pretorius et al., 2017c; de Waal et al., 2018).

Confocal analysis, as well as raw data of the clot analysis are shown in the Supplementary Material and in Figure 7. Control and PD platelet poor plasma clots, with markers illuminate amyloid fibrin(ogen) protein structure were imaged on a confocal microscope. Control clots display disperse signal. PD samples contain significantly greater amyloid-specific signal than control donors in all three channels: blue (*p* = 0.0002), red (*p* = 0.02), and green (< 0.0001).

## Discussion

In this paper, we show that in PD, there is a dysregulated systemic inflammatory biomarker profile (multiplex analysis), and that whole blood of these individuals are hypercoagulable (TEG analysis), with platelets hyperactivated (SEM analysis) and fibrin(ogen) taking on amyloid features (confocal assay). In the current paper, the most significant differences were noted in the HbA1c and HDL (pathology analysis); *R*-value, Alpha angle and TMRTG (TEG parameters); and IL-1α, IL-1β, IL-17A and TNFα (proinflammatory markers) (note that none of the parameters were significantly predictive of PD severity from the Hoehn and Yahr scale). Taken together, these results point to an inter-linked relationship between the hypercoagulability, inflammatory molecule presence, and platelet activation. Table 3 compares meta-analysis findings from Qin et al. (2016), with our results.

**TABLE 3:** Results from the current analysis, compared to meta-analysis by Qin et al. (2016).

**Measurement**	**Alignment with previous meta-analysis**	**Additional notes**
IL-6	Current paper does not show significance	*P* = 0.045 in meta-analysis
TNF-α	Agrees (significance)	
IL-1 β	Agrees (significance)	Not significant in ordinal model (see IL-1α note below)
CRP	Current paper does not show significance	*P* = 0.02 in meta-analysis
IL-10	Current paper does not show significance	*P* = 0.02 in current paper
IL-2	Not measured in current paper	
IFNγ	Agrees (Non-significance)	
IL-4	Agrees (Non-significance)	
IL-8	Agrees (Non-significance)	
IL-1α	Not measured in meta-analysis	Significant in ordinal model unlike IL-1β
IL-17A	Not measured in meta-analysis	

The pro-inflammatory profile of PD may also relate to blood clotting in various ways. These molecules may all act as outside-in signaling ligands (Durrant et al., 2017) that bind to platelet receptors, followed by inside-out signaling (Faull and Ginsberg, 1996) resulting in platelet dysfunction. The consequence after inflammatory molecule receptor binding is platelet activation, visible as platelet (hyper)activation, spreading and aggregation (or clumping). The subsequent platelet pathology, together with other changes in the hematological system such as anomalous fibrin(ogen) protein structure (discussed below) and RBC eryptosis [previously noted (Pretorius et al., 2018c)], all reflect the presence of systemic inflammation. Here, the inflammatory molecules in our panel that showed the most significance in PD, and particularly IL-1α, IL-1β, IL-17A, and TNF-α are all known to be dysregulated in cardiovascular disease and their presence in circulation might be linked to atherosclerosis (Libby, 2017; Wang et al., 2017).

### Platelet (Hyper) Activation in Parkinson’s Disease and Why They Might Be Targets for Upregulated, Circulating Cytokines

**FIGURE 8:**
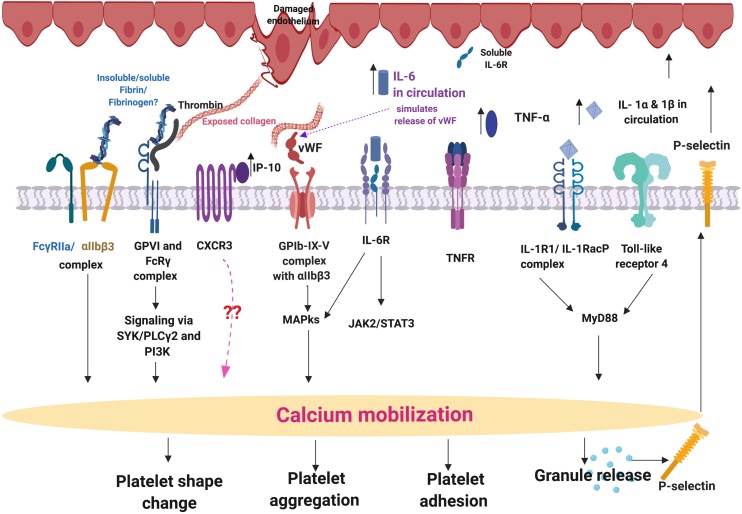
Simplified platelet signaling and receptor activation with main dysregulated molecules IL-1α, IL-1 β, TNF-α, and IL-17A. When inflammatory molecules are upregulated in circulation, they either cause direct endothelial damage (by binding to receptors on endothelial cells), or they may act as ligands that bind directly to platelet membrane receptors (Olumuyiwa-Akeredolu et al., 2019). When these inflammatory molecules disrupt endothelial cell structure, the endothelial cells release collagen and von Willebrand Factor (vWF). vWF is also a mediator of vascular inflammation (Gragnano et al., 2017), and it binds to exposed collagen and anchors platelets to the subendothelium (Du, 2007), causing platelet aggregation (Xu et al., 2016), and formation of a platelet plug (Jagadapillai et al., 2016). Both collagen and vWF act as platelet receptor ligands, causing platelet outside-in signaling, followed by inside-out signaling. Furthermore, collagen and vWB binding also result in signaling processes that cause a release of stored molecules that are present inside α- and dense granules of platelets, and may also include stored interleukins (e.g., IL-6 and IL-1β); further increasing the concentration of these inflammatory molecules in circulation (Olumuyiwa-Akeredolu et al., 2019). vWF binding is mediated by GpIbα (which is part of the GPIb-IX-V) and integrin (αIIbβ3 complex (Bryckaert et al., 2015). This αIIbβ3 receptor also binds fibrinogen and thrombin, and both these molecules and vWF work together to play critical roles in platelet activation and aggregation (Estevez and Du, 2017). Diagram created using BioRender (https://biorender.com/).

We seek to provide a possible explanation for the significant platelet activation that we observed by closely looking at our cytokine results, and particularly some of the most prominent dysregulated inflammatory markers. We focus here mainly on IL-1α, IL-1 β, IL-17A, and TNF-α, and appraised literature that previously linked upregulation of these molecules to platelet activation. These cytokines are all known to act as ligands to platelet receptors, which cause outside-in and inside-out platelet signaling See Figure 8 for a simplified diagram of such pathways receptor binding, as well as signaling.

IL-1α, IL-1β, IL-17A, and TNF-α are all significantly upregulated in our PD sample, and circulating TNF-α, IL-1, and IL-17 are also known to stimulate vWF release from damaged endothelial cells (Domingueti et al., 2016; Meiring et al., 2016; Owczarczyk-Saczonek and Placek, 2017). The IL-1 family of ligands and receptors are associated with both acute and chronic inflammation (Gabay et al., 2010; Dinarello, 2011), and IL-1α is an intracellular cytokine involved in various immune responses and inflammatory processes (Schett et al., 2016), that is known to be upregulated in cardiovascular diseases (Pfeiler et al., 2017). IL-1α has properties of both a cytokine and a transcription factor (Dinarello, 2006), and both IL-1α and IL-1β bind to the IL-1 receptor type 1 (IL-1RI), followed by recruitment of the co-receptor chain, the accessory protein, IL-1RAcP. A complex is formed consisting of IL-1RI, the ligand, IL-1α and the co-receptor (IL-1RAcP). This results in downstream signaling, involving the recruitment of the adaptor protein MyD88 to the Toll-IL-1 receptor domain. Platelets express IL-1R1, as well as Toll-like receptors, and these two receptors are known to be involved in platelet activation, platelet-leukocyte reciprocal activation, and immunopathology (Anselmo et al., 2016). Platelets also signal through the TLR4/MyD88- and cGMP/PKG-dependent pathway (Zhang et al., 2009), causing granule secretion followed by platelet activation and aggregation (Vallance et al., 2017). TNF-α, binds to two TNFα receptors present on platelets, TNFR1 and TNFR2, resulting in inside-out signaling and platelet (hyper)activation (Pignatelli et al., 2008). Platelets express a receptor for IL-17A, the IL-17R, receptor and the cytokine might facilitate their adhesion to damaged endothelium, as well as to other circulating leukocytes, ultimately leading to thrombus formation (Maione et al., 2011). Furthermore, IL-17A facilitates platelet function through the ERK2 signaling pathway (part of the MAPK pathway) and causes platelet aggregation (Zhang et al., 2012). IL-17A also promotes the exposure of α_*IIb*_β_3_ integrin, which provides more ligand binding site for fibrinogen *via* conformational change, and crosslinks the neighboring activated platelets which results in platelet aggregation (Zhang et al., 2012). These upregulated cytokines in our PD sample therefore could potentially be relate to the hyperactivated platelet ultrastructure shown in Figure 4.

### Amyloid Nature of Parkinson’s Disease Fibrin(ogen)

Previously, we have shown with ThT that the fibrin(ogen) protein structure in PD can become amyloid in nature, due to mis-folding of the protein (Pretorius et al., 2018c). It is well known that fibrinogen levels in PD is higher compared to healthy controls (Wong et al., 2010; Ton et al., 2012). In the current paper, we include two additional amyloid markers. Our results show enhanced amyloid-fluorescence as assessed by both AmyTracker 480 and 680 and this is confirmed by enhanced ThT fluorescent in our current PD samples. Our results suggest that in PD clots, fibrinogen polymerizes into a form with a greatly increased number of ß-sheets, reflecting amyloid formation. This important finding may describe a possible mechanism underlying some of the anomalous clotting formation and coagulopathies occurring in PD. It further emphasizes the systemic nature of PD, demonstrating pathological changes beyond the brain and extending to the circulation. Amyloid fibrin has also been observed in other diseases associated with inflammation and with known hematological abnormalities, including Type 2 Diabetes (Pretorius et al., 2017b, c) and

Alzheimer’s Disease (Pretorius et al., 2018a). Furthermore, an amyloid state may be induced experimentally by the addition of bacterial membrane products and iron (Pretorius et al., 2018b), as well as products of the acute phase response such as serum amyloid A (Page et al., 2019). The evidence provided here imply that the presence of (bacterial) inflammagen molecules, and the inflammatory state more broadly, are conditions that divert fibrinogen polymerization to an amyloid form, and indeed may be overarching (general) features of many chronic, inflammatory diseases (Kell and Pretorius, 2018a).

### The Presence of Bacterial Inflammagens in Parkinson’s Disease

*P gingivalis has* long been implicated in PD and periodontitis, and recently its protease (gingipain) was interrogated as a causative agent in AD, where the gingipain proteases was found in brain tissue from patients with AD (Dominy et al., 2019). These researchers also correlated these gingipain quantities within the brain tissue to the extent of tau and amyloid-β pathology. Furthermore, *P. gingivalis* has been found within atherosclerotic tissue of cardiovascular disease patients (Velsko et al., 2014; Olsen and Progulske-Fox, 2015; Atarbashi-Moghadam et al., 2018). Periodontal diseases are a well-known accompaniment to PD (Schwarz et al., 2006; Zlotnik et al., 2015; Kaur et al., 2016; Chen et al., 2017, 2018), however, the direct identification of *P. gingivalis* or its molecular signatures in circulation and/or brain tissue of PD patients has not previously been made.

Previous studies conducted on fibrinogen and plasma have shown that Rgp and Kgp increase thrombin time when compared to control samples (Imamura et al., 1995). Furthermore, the activation of other coagulation factors by gingipains have been established, including factor IX, X and prothrombin prothrombin (Imamura et al., 1997, 2001). Based on these observations, there seems to be a major disruption in the homeostatic control of the coagulation system/cascade when gingipain proteases are present. Here, we show that RgpA protease produced by *P. gingivalis* is present in PPP clots from our PD sample blood using polyclonal antibodies. We also confirmed that in healthy control PPP clots, less signal was observed compared to the PD clot samples (see Figure 6). In addition, we could detect RgpA with its fluorescent antibody in healthy control clots after adding recombinant protease to healthy control PPP. We have used a fluorescent purified fibrinogen model to show that LPS from *P. gingivalis* can cause hypercoagulability and that RgpA could hydrolyze fibrin(ogen) to such an extent that healthy control clot formation is visibly impaired. However, when both *P. gingivalis* LPS and RgpA are co-incubated, abnormal (hyperclottable) fibrin(ogen) is still visible. These results support our findings that in PD clots are more dense and hyperclottable (Pretorius et al., 2018c). It also supports our current TEG results that showed a hyperclottable clot phenotype in PD patients. Our preliminary results, where we identified RgpA with antibodies in plasma of PD individuals, is therefore an important finding for possible early identification of bacterial involvement in PD, and may lead to further research to clarify its role in this complex condition. However, our current experiments do not allow us to directly link the presence of inflammation (and dysregulated cytokines in circulation) and RgpA presence and blood of PD. An important consideration is that RgpA may hinder fibrinogen formation when present in blood, as seen with purified fibrinogen, however, its activity and resultant effect on coagulation may be altered in blood samples from patients, as various protease inhibitors and other RgpA targets reside within blood plasma. Albeit, with results shown in Figure 5, it can be concluded that RgpA has a diminishing effect on clot formation in terms of purified fibrinogen catalyzed by thrombin, but a decreased effect when co-incubated with LPS.

These results are of particular importance when it is noted that bacterial involvement might play a role in both the development and even progression of PD, and specifically, circulating bacterial inflammagens such as LPS have been implicated (Tufekci et al., 2011; De Chiara et al., 2012; Potgieter et al., 2015; Friedland and Chapman, 2017). We have also suggested that LPS may both maintain systemic inflammation, as well as the disease etiology itself in PD (but also in other inflammatory diseases like type 2 diabetes, pre-eclampsia, sepsis, rheumatoid arthritis and Alzheimer’s disease, where LPS presence has been implicated in the etiology of the condition) (Kell and Kenny, 2016; Pretorius et al., 2016a, b, 2017a, b, c; Kell and Pretorius, 2018b). Indeed in 2018, we showed that LPS from *E. coli* could be identified with fluorescent LPS *E. coli* antibodies in clots of PD, type 2 diabetes and AD (de Waal et al., 2018). There is therefore mounting evidence that PD might have a bacterial involvement, that in part drives the etiology of the condition through endotoxins (and exotoxins) as potent bacterial inducers of cytokines (Cavaillon, 2018).

We conclude by proposing that our results strongly support a systemic inflammatory and hypercoagulable pathology fueled by a bacterial presence, and serves as a preliminary study showing a role of *P. gingivalis* LPS and gingipain protease in abnormal blood clotting observed in our PD sample. A future strategy would be to identify the extent to which this bacterium might contribute to PD pathology, or if there are any specific links, e.g., a link with the presence of α-synuclein and auto/xenophagy (El-Awady et al., 2015; Cerri and Blandini, 2018). Furthermore, our finding that gingipain antibody signal was detected in clots from our PD samples but not the healthy controls, emphasizes the possibility of this bacterium having a role in PD pathology. We have discussed research that pointed to the fact that bacteria, more generally, are implicated in PD etiology, and here we note the possible involvement of *P. gingivalis*, specifically. Taking these strong associations into consideration, and extrapolating in both a neurological and cardiovascular context, it is plausible to believe that the entry, dissemination and infection of this bacterium and its virulent machinery in a systemic manner may be an etiological and/or driving factor for disease worth investigating further.

## Data Availability

The raw data files are accessible at: https://1drv.ms/f/s!AgoCOmY3bkKHibs-vg0EUq3N5SogfA. All datasets generated for this study are included in the manuscript and/or the Supplementary Files.

## Ethics Statement

The studies involving human participants were reviewed and approved by Health Research Ethics Committee (HREC) of Stellenbosch University (South Africa) and the Western Cape Department of Health. The patients/participants provided their written informed consent to participate in this study.

## Author Contributions

BA: patient blood collection and preparation of blood samples, TEG and 20-plex analysis, and statistics. TN: statistics and editing of the manuscript. JN: gingipain experiments. MP: amyloid assay and editing of the manuscript. TR: all correlation analysis and plots. JC: clinician. DK: edit the manuscript and co-corresponding author. EP: study leader, writing of the manuscript, and co-corresponding author. All authors reviewed the manuscript.

## Conflict of Interest Statement

The authors declare that the research was conducted in the absence of any commercial or financial relationships that could be construed as a potential conflict of interest.

## Abbreviations

EDTA: ethylenediaminetetraacetic acid
ERK2: extracellular signal–regulated kinase 2
E-Selectin: E-Selectin
GM-CSF: Granulocyte-macrophage colony-stimulating factor
HbA1c: glycosylated hemoglobin
HDL: high-density lipoprotein
IFN- α: interferon-alpha
IFN γ: interferon-gamma
IL-1 β: interleukin-1 beta
IL-1 α: interleukin-1 alpha
IL-10: interleukin-10
IL-12p70: interleukin-12p70
IL-13: interleukin-13
IL-17A: interleukin-17A
IL-4: interleukin-4
IL-6: interleukin-6
IL-8: interleukin-8
IP-10: interferon gamma-induced protein-10
LDL: low-density lipoprotein
LPS: lipopolysaccharide
LTA: lipoteichoic acid
MCP-1: monocyte chemoattractant protein-1
MIP-1 α: macrophage inflammatory protein-1 alpha
MIP-1 β: macrophage inflammatory protein-1 beta
NBF: neutral buffered formalin
PBS: phosphate buffered saline
PD: Parkinson’s disease
PPP: platelet poor plasma
P-Selectin: P-Selectin
RBC: red blood cell
RgpA: gingipain R1 protease
sICAM-1: soluble intercellular adhesion molecule-1
SNpc: substantia nigra pars compacta
SST: serum separating tubes
TC: total cholesterol
TEG: thromboelastography
TG: triglyceride
ThT: thioflavin T
TNF- α: tumor necrosis factor-alpha
WB: whole blood.
