# Supplementary material for: Parkinson’s Disease: A Systemic Inflammatory Disease Accompanied by Bacterial Inflammagens
Source: Front Aging Neurosci. 2019 Aug 27;11:210. doi: 10.3389/fnagi.2019.00210 (PMC6718721; doi:10.3389/fnagi.2019.00210)
Supplement: Supplementary file 1 [file Data_Sheet_1.pdf]

SUPPLEMENTARY FIGURES AND TABLES

Overview diagram: Figure 1 An overview summarizing the objectives of this manuscript. **(**1) Whole blood (WB) and platelet poor plasma (PPP) samples from Parkinson's disease individuals were used as our disease model for systemic inflammation (2), and compared to healthy whole blood and plasma samples. (3) Concentrations of various circulating inflammatory markers (multiplex and ELIZA analyses) was determined, as well as blood clotting propensity (thromboelastography). Platelet ultrastructure was also shown. (4) The presence of the bacterial protease gingipain R1 was detected in PPP clots, using antibodies. Recombinant gingipain R1 was also be added to fluorescent (ALEXA488) purified fibrinogen to determine its effect on clot formation. Finally, we confirmed amyloid formation in fibrin(ogen) protein, using novel amyloid detecting fluorescent markers (Amytrackers™). We then propose how the above influences the nature of hypercoagulation (5), as well as the innate immune (dys)function in PD (6). Diagram created using BioRender (https://biorender.com/).

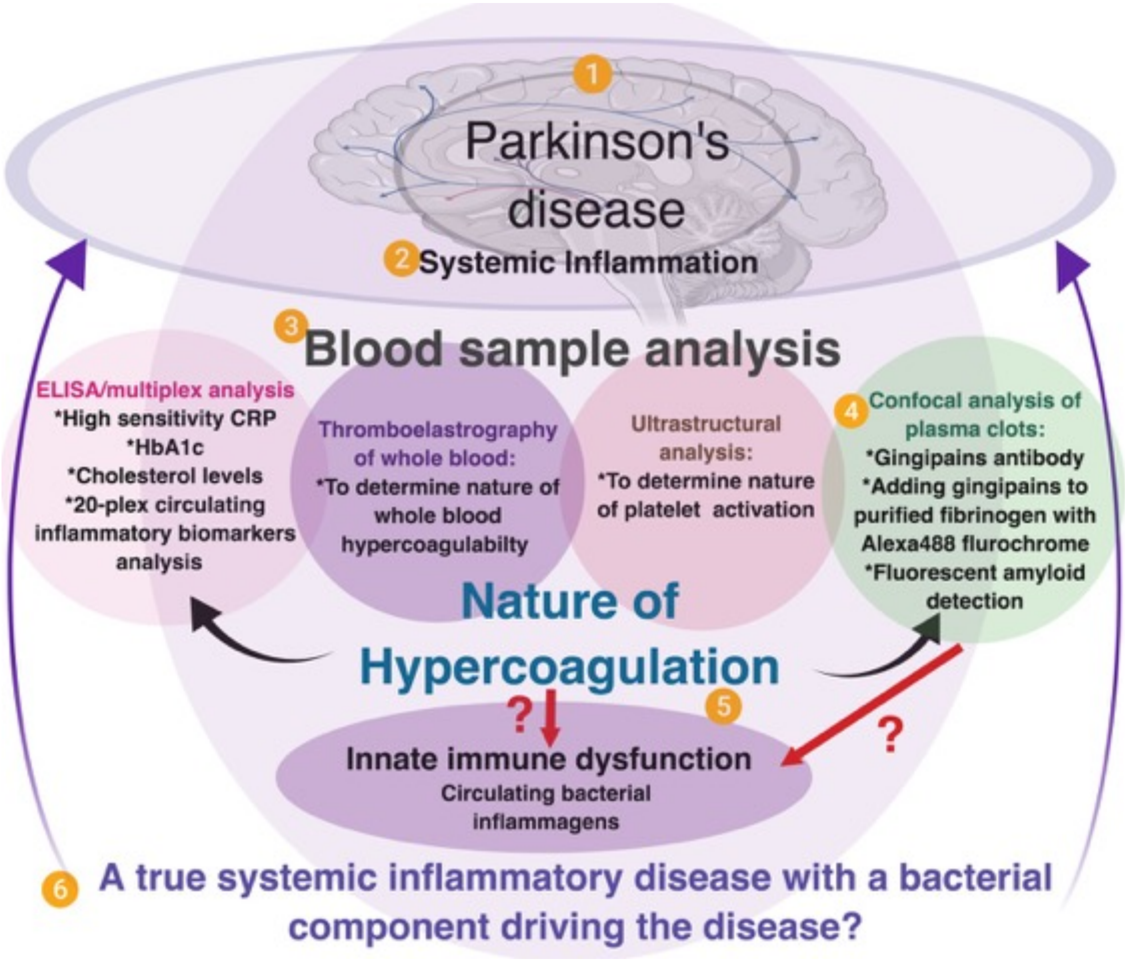

Figure 2 PCA Exploratory Analysis: **(A)** PCA Bi-plot with colored ellipses for Control and PD populations and **(B)** corresponding Screeplot showing the distribution of variance across principle components.

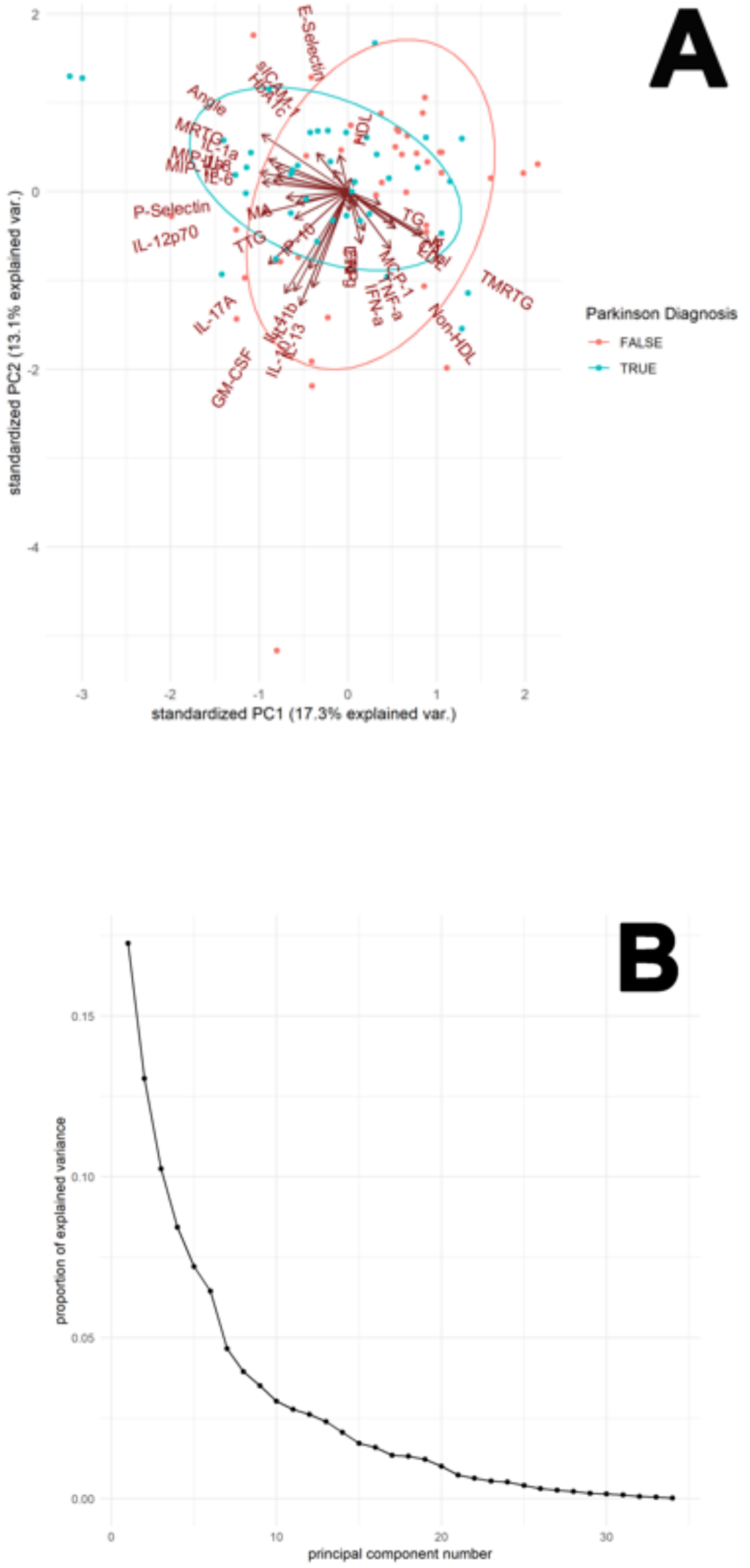

Supplementary table 1:
The Hoehn and Yahr staging system in Parkinson's Disease

Stage	Criteria
**Stage 0**	No signs of disease
**Stage 1**	Unilateral disease only
**Stage 1.5**	Unilateral disease plus axial involvement
**Stage 2**	Bilateral disease, without impairment of balance
**Stage 2.5**	Mild bilateral disease, with recovery on pull test
**Stage 3**	Mild to moderate bilateral disease; some postural instability; physically independent
**Stage 4**	Severe disability; still able to walk or stand unassisted
**Stage 5**	Wheel chair bound or bedridden unless aided

Supplementary table 2:
Parkinson's disease sample demographics, Hoehn and Yahr stages, as well as medication.

atient ID PD	Age	Gender	Hoehn and Yahr scale	PD Medication
PD 1	58	Male	2	Cabilev
PD 2	69	Female	2	Cabilev,Rivotril,Sinemet,Hydrochlorothiazide
PD 3	53	Male	2	Cabilev,Sinemet
PD 4	68	Male	3	Levodopa
PD 5	59	Female	2	Cabilev,Donperidone,Risperidone
PD 6	66	Male	3	Cabilev,Requip,Sinemet
PD 7	59	Male	2	Cabilev
PD 8	59	Male	3	Cabilev,Fluoxetine,Fludrocortisone
PD 9	59	Male	2	Cabilev
PD 10	69	Female	2	Cabilev,Amlodipine
PD 11	68	Female	3	Cabilev,Aspirin,Metformin
PD 12	75	Male	2.5	Cabilev
PD 13	64	Male	2.5	Cabilev,Ropinirole
PD 14	65	Male	1	Cabilev,Pexola
PD 15	64	Female	1	Cabilev,Clonazepam,Metformin,Enalapril
PD 16	66	Female	4	Cabilev,Ropinirole,Amlodipine,Clonazepam,Oxazepam
PD 17	64	Male	1	Cabilev,Enalapril
PD 18	63	Male	2.5	Cabilev,Rivotril
PD 19	64	Male	4	Cabilev,Rivotril,Pexola,Orphenadrine
PD20	72	Female	3	Cabilev,Donperidone
PD 21	87	Male	2	Cabilev,Levodopa,Metformin,Aspirin,Atenolol
PD 22	70	Male	2	Cabilev,Clonazepam,Sinemet,Enalapril
PD 23	62	Female	3	Cabilev,Pexola
PD 24	71	Female	4	Cabilev,Sinemet,Aspirin
PD 25	64	Male	1.5	Cabilev
PD 26	63	Male	3	Cabilev,Clonazepam,Pexola,Rivotril, Amlodipine, Hydrochlorothiazide
PD 27	77	Female	2	Cabilev,Clonazepam
PD 28	71	Female	1	Cabilev,Sinemet,Amlodipine,Aspirin, Metformin
PD 29	73	Male	2	Newly diagnosed, Metformin
PD 30	87	Male	3	Cabilev,Aspirin,Simvastatin,Metformin
PD 31	72	Female	5	Cabilev,Insulin
PD 32	65	Male	2	Cabilev,Amlodipine
PD 33	81	Male	2.5	Cabilev
PD 34	52	Male	1	Cabilev,Insulin
PD 35	60	female	2.5	Cabilev,Rivotril,Orphenadrine
PD 36	72	Male	1	Cabilev,Pexola,Enalapril

PD 37	79	Male	2.5	Cabilev
PD 38	83	Female	2	Cabilev,Rivotril
PD 39	59	Female	1	Orphenadrine,Clonazepam
PD 40	84	Male	3	Cabilev
